# WISP-3 promotes tumor-monocyte adhesion through a MEK/ERK-dependent miR-12131/ICAM-4 axis in lung adenocarcinoma

**DOI:** 10.7150/ijms.136597

**Published:** 2026-07-13

**Authors:** Chia-Chia Chao, Syuan-Ling Lin, Yu-Chen Chen, Ching-Yuan Cheng, En-Ming Chang, Chih-Hsin Tang, Chih-Yang Lin

**Affiliations:** 1 Department of Respiratory Therapy, Fu Jen Catholic University, New Taipei City, Taiwan.; 2 Center for Health Research and Innovation, Fu Jen Catholic University, New Taipei City, Taiwan.; 3 Translational Medicine Research Center, China Medical University Hospital, Taichung, Taiwan; 4 Graduate Institute of Biomedical Sciences, China Medical University, Taichung, Taiwan; 5 Translational Medicine Center, Shin Kong Wu Ho-Su Memorial Hospital, Taipei, Taiwan; 6 Division of Chest Medicine, Shin Kong Wu Ho-Su Memorial Hospital, Taipei, Taiwan; 7 Department of Respiratory Care, Shin Kong Wu Ho-Su Memorial Hospital, Taipei City, Taiwan; 8 Department of Pharmacology, School of Medicine, China Medical University, Taichung, Taiwan; 9 Department of Medical Laboratory Science and Biotechnology, Asia University, Taichung, Taiwan; 10 Chinese Medicine Research Center, China Medical University, Taichung, Taiwan; 11 Department of Medical Research, China Medical University Hospital, China Medical University, Taichung 404327, Taiwan.

**Keywords:** WISP-3, ICAM-4, miR-12131, monocyte adhesion, lung adenocarcinoma

## Abstract

Tumor-immune cell interactions critically contribute to the progression of non-small cell lung cancer (NSCLC). In this study, we investigated the role of WNT1-inducible signaling pathway protein 3 (WISP-3) in regulating tumor cell adhesion and the underlying molecular mechanisms in lung adenocarcinoma cells. Treatment with recombinant WISP-3 significantly increased intercellular adhesion molecule-4 (ICAM-4) expression at both mRNA and protein levels in A549 and H1299 cells in a dose-dependent manner. Consistently, WISP-3 enhanced tumor-monocyte adhesion, indicating its involvement in tumor-immune cell interactions. Mechanistically, WISP-3 stimulated rapid activation of the MEK/ERK signaling cascade, as demonstrated by increased phosphorylation of MEK and ERK. Pharmacological inhibition of MEK using PD98059 or U0126, as well as direct inhibition of ERK with SCH772984, markedly attenuated WISP-3-induced ICAM-4 expression and THP-1 adhesion. These findings were further supported by siRNA-mediated knockdown of MEK or ERK, confirming the essential role of this pathway. In addition, WISP-3 suppressed the expression of hsa-miR-12131, which was identified as a negative regulator of ICAM-4. Restoration of hsa-miR-12131 significantly reduced ICAM-4 expression and impaired tumor-monocyte adhesion, indicating that miR-12131 functions downstream of MEK/ERK signaling. Collectively, these results demonstrate that WISP-3 promotes ICAM-4-dependent monocyte adhesion through activation of the MEK/ERK pathway and subsequent suppression of hsa-miR-12131. This WISP-3/MEK/ERK/miR-12131/ICAM-4 axis provides new insight into tumor-immune interactions in NSCLC and highlights potential therapeutic targets.

## Introduction

Lung cancer remains the leading cause of cancer-related mortality worldwide, with lung adenocarcinoma (LUAD) representing the most prevalent subtype of non-small cell lung cancer (NSCLC) 1. Despite advances in targeted therapy and immunotherapy, patient outcomes remain unsatisfactory, largely due to metastasis and tumor recurrence 2. Increasing evidence indicates that tumor progression is not solely driven by intrinsic properties of cancer cells but is also critically regulated by the tumor microenvironment (TME) 3, 4. Within this context, monocytes and tumor-associated macrophages (TAMs) have emerged as key components that contribute to tumor progression, immune evasion, and metastasis 5, 6. These cells originate from circulating monocytes, which are recruited to tumor sites and subsequently differentiate into TAMs, thereby promoting tumor growth and suppressing anti-tumor immunity 5. Given their central role in tumor-immune interactions, cell adhesion molecules are essential mediators that facilitate not only physical cell-cell contact but also signaling events that regulate immune cell behavior 7. Among the intercellular adhesion molecule (ICAM) family members, ICAM-1 has been extensively studied in cancer progression and immune cell recruitment, whereas the biological function of ICAM-4 in solid tumors remains largely unexplored. Because adhesion molecules can influence interactions between tumor cells and immune cells within the tumor microenvironment, identifying less-characterized adhesion regulators may provide additional insights into tumor-immune communication. However, the molecular mechanisms governing tumor-monocyte adhesion in LUAD remain poorly understood.

The CCN (Cyr61/CTGF/NOV) family of matricellular proteins has emerged as an important group of regulators that integrate extracellular cues with intracellular signaling, thereby modulating cell-matrix and cell-cell interactions in cancer 8, 9. Among these proteins, WNT1-inducible signaling pathway protein 3 (WISP-3/CCN6) has been implicated in diverse biological processes, including extracellular matrix remodeling, cell proliferation, and tumor progression 8, 10. Although WISP-3 has been reported to exhibit context-dependent functions across different cancer types, accumulating evidence suggests that CCN family proteins participate in the regulation of extracellular matrix remodeling, cell adhesion, and communication between tumor cells and stromal components within the tumor microenvironment 11. Nevertheless, the role of WISP-3 in regulating adhesion-associated molecules and tumor-immune interactions remains poorly defined. Whether WISP-3 contributes to tumor-monocyte communication in lung adenocarcinoma has not been clearly elucidated.

The mitogen-activated protein kinase (MAPK) pathway, especially the MEK/ERK cascade, represents a central signaling axis that transduces extracellular stimuli into transcriptional responses controlling cell proliferation, survival, and differentiation 12, 13. Aberrant activation of MEK/ERK signaling is frequently observed in non-small cell lung cancer and has been closely associated with tumor progression, metastasis, and therapeutic resistance 12. Beyond its role in transcriptional regulation, accumulating evidence suggests that MAPK signaling can also influence post-transcriptional regulatory networks, including the modulation of microRNA (miRNA) expression, thereby linking extracellular signaling pathways to fine-tuning of gene expression programs 14, 15. These observations raise the possibility that MEK/ERK signaling may regulate adhesion-associated molecules through miRNA-dependent mechanisms.

MicroRNAs are small non-coding RNAs that regulate gene expression by targeting messenger RNAs and have been recognized as key regulators of cancer development and tumor-microenvironment interactions 15. In lung cancer, miRNAs have been shown to control a wide range of biological processes, including cell proliferation, invasion, metastasis, and immune modulation 16, 17. Notably, recent studies highlight that miRNAs can act as critical intermediates connecting oncogenic signaling pathways to specific downstream effectors 18, 19. However, despite extensive characterization of several canonical miRNAs, the functional roles of many less-studied miRNAs remain largely unexplored, particularly in the context of tumor-immune interactions.

Based on these observations, we sought to investigate whether WISP-3 contributes to tumor-monocyte adhesion in lung adenocarcinoma and to define the underlying molecular mechanisms. Specifically, we examined whether WISP-3 regulates adhesion-associated molecules through MEK/ERK signaling and miRNA-mediated pathways. Our findings identify a signaling axis involving WISP-3, MEK/ERK activation, miR-12131 suppression, and ICAM-4 upregulation that contributes to enhanced tumor-monocyte adhesion in LUAD.

## Material and Methods

### Reagents and inhibitors

Recombinant human WISP-3 protein was purchased from PeproTech (Rocky Hill, NJ, USA) and reconstituted according to the manufacturer's instructions. Cells were treated with WISP-3 at the indicated concentrations (0-100 ng/mL) for the specified durations. The MEK inhibitors PD98059 and U0126, as well as the ERK inhibitor SCH772984, were obtained from MedChemExpress (Monmouth Junction, NJ, USA) and dissolved in dimethyl sulfoxide (DMSO; Sigma-Aldrich, St. Louis, MO, USA). For inhibitor studies, cells were pretreated with PD98059, U0126, or SCH772984 for 30 min prior to WISP-3 stimulation. Control cells received an equivalent volume of DMSO. All other chemicals were obtained from Sigma-Aldrich (St. Louis, MO, USA).

### Cell culture

The human lung adenocarcinoma cell lines A549 and H1299 were obtained from American Type Culture Collection (ATCC, Manassas, VA, USA). Cells were maintained in RPMI-1640 medium supplemented with 10% fetal bovine serum (FBS) and 1% penicillin-streptomycin (Gibco-BRL Life Technologies, Grand Island, NY, USA). Cells were incubated at 37°C in a humidified atmosphere containing 5% CO₂. For WISP-3 stimulation experiments, A549 and H1299 cells were washed with PBS and cultured overnight in serum-free medium before treatment with recombinant human WISP-3 at the indicated concentrations and incubation times. The human monocytic cell line THP-1 was cultured in RPMI-1640 medium supplemented with 10% FBS under the same conditions.

### miRNA target prediction

Potential miRNAs targeting ICAM-4 were predicted using the miRWalk database. Candidate miRNAs were selected based on predicted binding sites within the 3′ untranslated region (3′UTR) of ICAM4. To improve prediction confidence, candidate miRNAs were further filtered using the miRDB database. Based on these analyses, hsa-miR-2355-5p, hsa-miR-6749-3p, and hsa-miR-12131 were selected for subsequent validation.

### siRNA and miRNA transfection

Small interfering RNAs (siRNAs) targeting MEK and ERK, as well as a non-targeting control siRNA, were purchased from Santa Cruz Biotechnology (Santa Cruz, CA, USA). Synthetic miR-12131 mimics and negative control mimics were obtained from AllBio RNA system (AllBio, Taichung, Taiwan). Cells were transfected with siRNAs or miRNA mimics at a final concentration of 50 nM using Lipofectamine RNAiMAX (Invitrogen, Carlsbad, CA, USA) according to the manufacturer's instructions. Briefly, siRNAs or miRNA mimics and Lipofectamine RNAiMAX were separately diluted in serum-free medium and incubated for 5 min at room temperature. The diluted Lipofectamine RNAiMAX was then mixed with diluted siRNAs or miRNA mimics and allowed to form transfection complexes for 20 min. The complexes were subsequently added to cells and incubated overnight. Cells were incubated for 24 h post-transfection before subsequent treatments or assays.

### THP-1 monocyte adhesion assay

Tumor cell adhesion assays were performed using A549 and H1299 cells as adherent cells and THP-1 cells as monocytes. THP-1 cells were labeled with BCECF-AM for 30 min at 37°C according to the manufacturer's instructions and washed with PBS prior to use. A549 and H1299 cells were seeded and treated with WISP-3 at the indicated concentrations or under inhibitor/siRNA/mimic conditions. After treatment, labeled THP-1 cells were added and co-incubated for 1 h. Non-adherent THP-1 cells were removed by gently washing the wells three times with PBS. Adherent THP-1 cells were visualized under a fluorescence microscope and quantified by counting adherent cells from five randomly selected fields per well. Adhesion activity was expressed as a percentage relative to the control group.

### RNA extraction and quantitative real-time PCR (RT-qPCR)

Cells were transfected or pretreated as described above, followed by WISP-3 stimulation for 24 h prior to RNA extraction. Total RNA was isolated using the easy-BLUE™ Total RNA Extraction Kit (iNtRON Biotechnology, Korea). For mRNA analysis, complementary DNA (cDNA) was synthesized using the Magic RT Mastermix cDNA synthesis kit (Biogenesis, Taiwan). RT-qPCR was performed using PowerUp™ SYBR™ Green Master Mix (Applied Biosystems, Thermo Fisher Scientific, USA) on a QuantStudio™ 1 Real-Time PCR System (Applied Biosystems, USA). Gene expression levels were normalized to GAPDH and calculated using the 2^-ΔΔCt method. For miRNA analysis, reverse transcription was performed using the Mir-X™ miRNA First-Strand Synthesis Kit (Takara Bio, Japan). miRNA expression levels were quantified by qPCR and normalized to U6 small nuclear RNA.

### Western blot analysis

Cells were transfected with siRNAs or miRNA mimics for 24 h or pretreated with inhibitors for 30 min, followed by WISP-3 stimulation for the indicated time points. Cells were lysed in RIPA buffer supplemented with protease and phosphatase inhibitors (Sigma-Aldrich, St. Louis, MO, USA). Protein concentrations were determined using a BCA protein assay kit (Thermo Fisher Scientific, Waltham, MA, USA). Equal amounts of protein were separated by SDS-PAGE and transferred onto PVDF membranes (Merck Millipore, Burlington, MA, USA). Membranes were blocked with 5% non-fat milk and incubated with primary antibodies against ICAM-4 (GeneTex, GTX51807, 1:1000), MEK1 (Santa Cruz Biotechnology, sc-6250, 1:1000), phospho-MEK1/2 (Ser221; Cell Signaling Technology, #2338, 1:1000), ERK2 (Santa Cruz Biotechnology, sc-1647, 1:1000), phospho-ERK1/2 (Thr202/Tyr204; Cell Signaling Technology, #4370, 1:1000), and β-actin (Thermo Fisher Scientific, MA5-15739, 1:10000), followed by HRP-conjugated secondary antibodies. Signals were detected using an enhanced chemiluminescence (ECL) system and visualized using a Fujifilm LAS-3000 imaging system. Band intensities were quantified using UN-SCAN-IT gel version 6.1 (Silk Scientific, Orem, UT, USA) and normalized to β-actin or total protein levels as indicated.

### Statistical analysis

All experiments were performed in at least three independent biological replicates. Data are presented as mean ± S.D. Statistical comparisons between two groups were performed using Student's t-test, whereas multiple group comparisons were analyzed using one-way ANOVA followed by Tukey's multiple comparisons test. A p value < 0.05 was considered statistically significant.

## Results

### WISP-3 enhances tumor-monocyte adhesion through selective induction of ICAM-4

**Figure 1:**
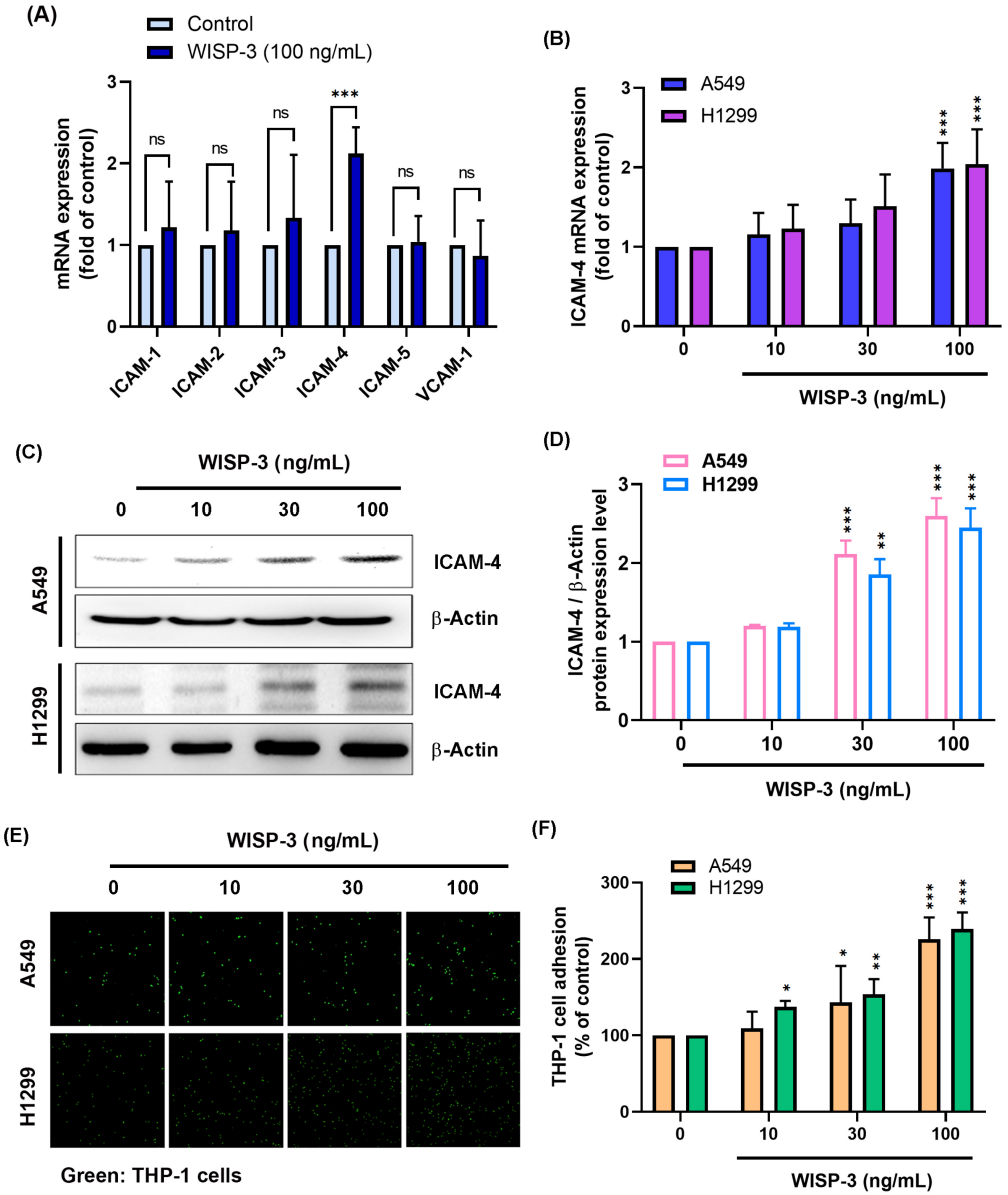
** WISP-3 selectively induces ICAM-4 expression and promotes tumor-monocyte adhesion in lung adenocarcinoma cells.** (A) H1299 cells were treated with WISP-3 (100 ng/mL), and the mRNA expression of ICAM family members and VCAM-1 was analyzed by qPCR. (B) ICAM-4 mRNA expression was measured by qPCR in A549 and H1299 cells treated with WISP-3 (0, 10, 30, or 100 ng/mL) for 24 h. (C&D) ICAM-4 protein expression was analyzed by western blot and quantified following treatment with WISP-3 (0, 10, 30, or 100 ng/mL) for 24 h. (E&F) Tumor-monocyte adhesion was assessed using a fluorescence-based adhesion assay following treatment of A549 and H1299 cells with WISP-3 and quantified. Data are presented as mean ± SD from at least three independent experiments. Statistical significance was defined as *P < 0.05, **P < 0.01, and ***P < 0.001 compared with control.

Given that WISP-3 has been implicated in the regulation of tumor-microenvironment interactions, particularly processes involving cell adhesion, we first examined whether WISP-3 modulates the expression of adhesion-associated molecules in lung adenocarcinoma cells. H1299 cells were treated with recombinant WISP-3, and the mRNA expression of ICAM family members was analyzed. WISP-3 had no significant effect on the expression of ICAM-1, ICAM-2, ICAM-3, ICAM-5, or VCAM-1, whereas ICAM-4 expression was markedly increased (Fig. 1A). A dose-dependent increase in ICAM-4 mRNA expression was subsequently confirmed in both A549 and H1299 cells following WISP-3 stimulation (Fig. 1B). Consistent with the qPCR results, western blot analysis demonstrated that WISP-3 also elevated ICAM-4 protein expression in both cell lines in a concentration-dependent manner (Fig. 1C&D). Because ICAM-4 is known to mediate cell-cell adhesion, we next evaluated whether WISP-3 influences tumor-monocyte interactions. Fluorescence-based adhesion assays revealed that WISP-3 significantly increased THP-1 monocyte adhesion to both A549 and H1299 cells in a dose-dependent manner (Fig. 1E&F), indicating that WISP-3 enhances monocyte adhesion in parallel with ICAM-4 upregulation.

### MEK signaling is required for WISP-3-mediated ICAM-4 expression and tumor-monocyte adhesion

**Figure 2:**
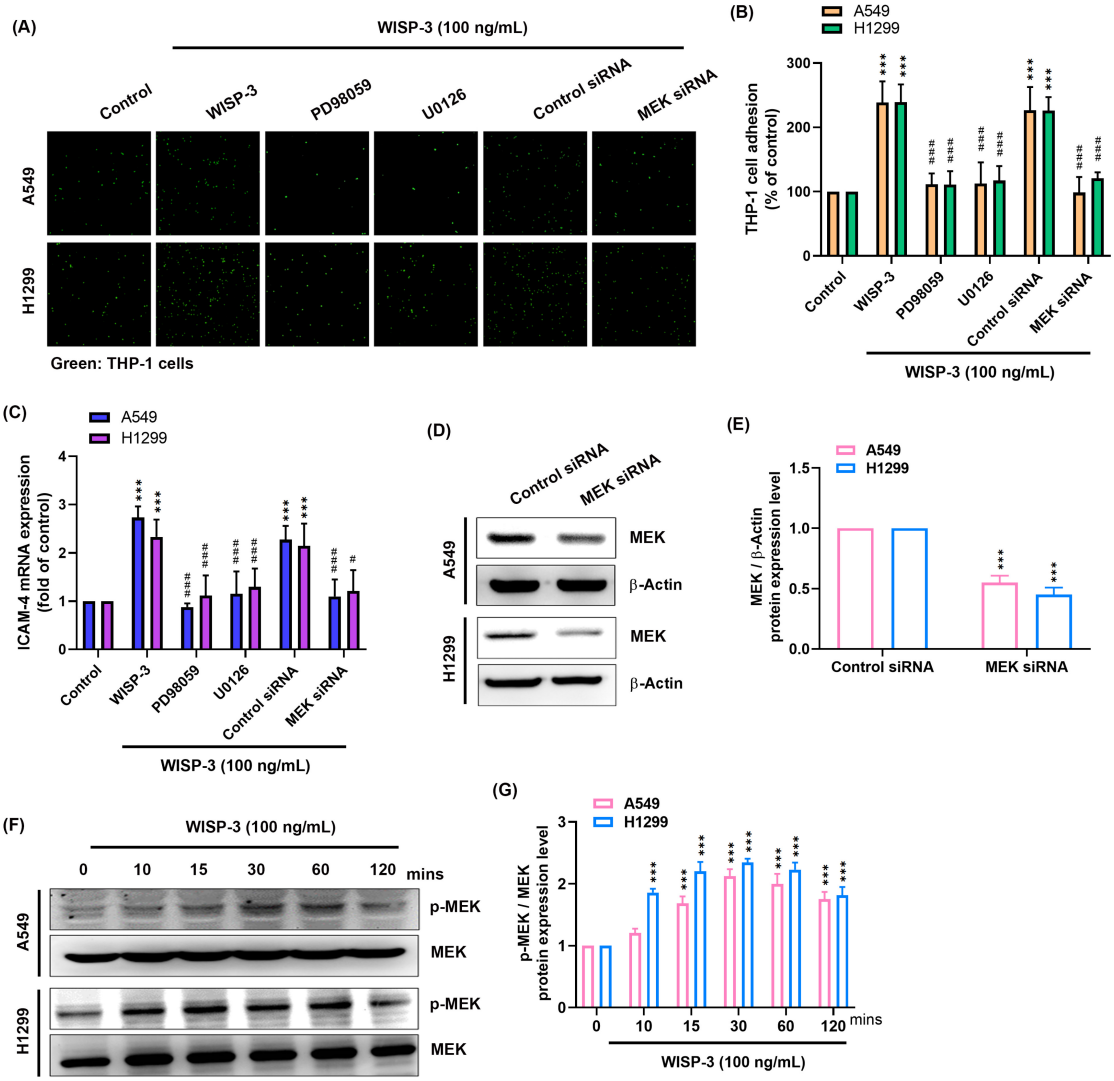
** MEK signaling mediates WISP-3-induced ICAM-4 expression and tumor-monocyte adhesion in lung adenocarcinoma cells.** (A&B) Tumor-monocyte adhesion was assessed after pretreatment with PD98059 (10 μM) or U0126 (10 μM) for 30 min, or transfection with control or MEK siRNA for 24 h, followed by WISP-3 stimulation (100 ng/mL, 24 h), and quantified. (C) ICAM-4 mRNA expression was measured by qPCR after pretreatment with PD98059 (10 μM) or U0126 (10 μM) for 30 min, or transfection with control or MEK siRNA for 24 h, followed by WISP-3 stimulation (100 ng/mL) for 24 h. (D&E) MEK protein expression following transfection with control or MEK siRNA was analyzed by western blot and quantified. (F&G) MEK phosphorylation was analyzed by western blot after WISP-3 stimulation (100 ng/mL) for the indicated times and quantified. Data are presented as mean ± SD from at least three independent experiments. Statistical significance was defined as *P < 0.05, **P < 0.01, and ***P < 0.001 versus control; #P < 0.05, ##P < 0.01, and ###P < 0.001 versus WISP-3-treated group.

Given that activation of MAPK signaling pathways is commonly involved in the regulation of adhesion-related gene expression, we next examined whether MEK signaling contributes to WISP-3-induced ICAM-4 expression and monocyte adhesion. A549 and H1299 cells were pretreated with the MEK inhibitors PD98059 or U0126 prior to WISP-3 stimulation. Inhibition of MEK markedly reduced WISP-3-induced THP-1 adhesion in both cell lines (Fig. 2A&B). Consistently, WISP-3-induced ICAM-4 mRNA expression was significantly attenuated in the presence of PD98059 or U0126 (Fig. 2C). To further validate the involvement of MEK, siRNA-mediated knockdown was performed, and efficient reduction of MEK protein levels was confirmed by western blot analysis (Fig. 2D&E). MEK depletion similarly suppressed both ICAM-4 expression and THP-1 adhesion induced by WISP-3 (Fig. 2A-C). In addition, WISP-3 stimulation led to a rapid increase in MEK phosphorylation in both A549 and H1299 cells, while total MEK expression remained largely unchanged (Fig. 2F). These data indicate that MEK activation is involved in mediating the effects of WISP-3 on ICAM-4 expression and monocyte adhesion.

### ERK activation mediates WISP-3-induced ICAM-4 expression and tumor-monocyte adhesion

**Figure 3:**
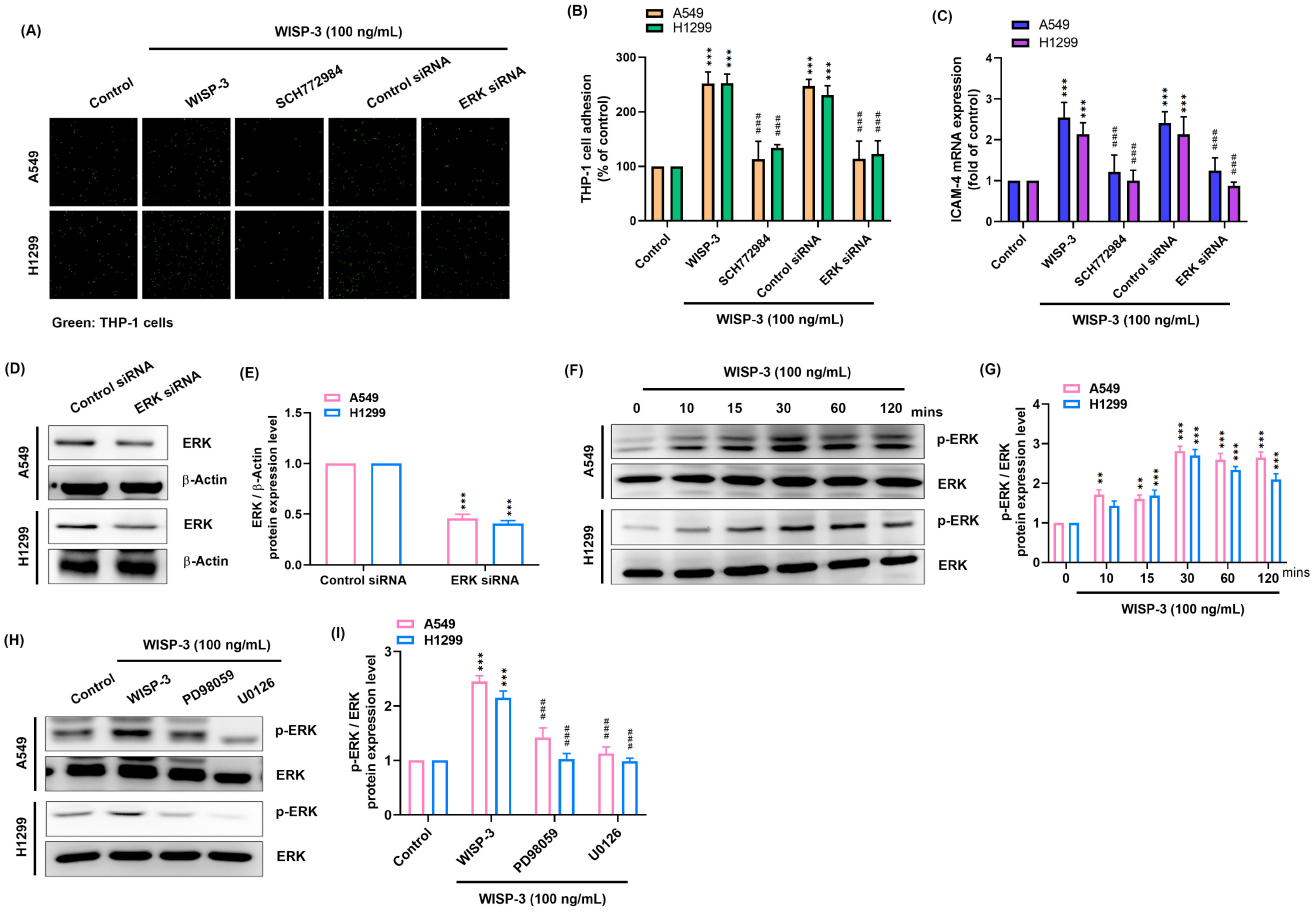
** Role of ERK activation in WISP-3-induced ICAM-4 expression and monocyte adhesion.** (A&B) Tumor-monocyte adhesion was assessed after pretreatment with SCH772984 (10 μM) for 30 min, or transfection with control or ERK siRNA for 24 h, followed by WISP-3 stimulation (100 ng/mL) for 24 h, and quantified. (C) ICAM-4 mRNA expression was measured by qPCR after pretreatment with SCH772984 (10 μM) for 30 min, or transfection with control or ERK siRNA for 24 h, followed by WISP-3 stimulation (100 ng/mL) for 24 h. (D&E) ERK protein expression following transfection with control or ERK siRNA was analyzed by western blot and quantified. (F&G) ERK phosphorylation was analyzed by western blot after WISP-3 stimulation (100 ng/mL) for the indicated time and quantified. Data are presented as mean ± SD from at least three independent experiments. Statistical significance was defined as *P < 0.05, **P < 0.01, and ***P < 0.001 versus control; #P < 0.05, ##P < 0.01, and ###P < 0.001 versus WISP-3-treated group.

Given that ERK is a key downstream effector of MEK signaling, we next examined whether ERK mediates the effects of WISP-3 on ICAM-4 expression and monocyte adhesion. Treatment with the ERK inhibitor SCH772984 significantly reduced WISP-3-induced THP-1 adhesion in both A549 and H1299 cells (Fig. 3A&B). In parallel, WISP-3-induced ICAM-4 mRNA expression was markedly attenuated upon ERK inhibition (Fig. 3C). To further validate the role of ERK, siRNA-mediated knockdown was performed, and efficient suppression of ERK protein expression was confirmed (Fig. 3D&E). Consistently, ERK depletion reduced both ICAM-4 expression and monocyte adhesion in response to WISP-3 (Fig. 3A-C). WISP-3 stimulation induced rapid ERK phosphorylation without affecting total ERK levels (Fig. 3F). Notably, inhibition of MEK using PD98059 or U0126 markedly suppressed WISP-3-induced ERK phosphorylation (Fig. 3G&H), demonstrating that ERK activation is dependent on upstream MEK signaling. These data indicate that ERK functions downstream of MEK to mediate WISP-3-induced ICAM-4 expression and monocyte adhesion.

### WISP-3 suppresses miR-12131 to enhance ICAM-4 expression and tumor-monocyte adhesion in lung adenocarcinoma cells

**Figure 4:**
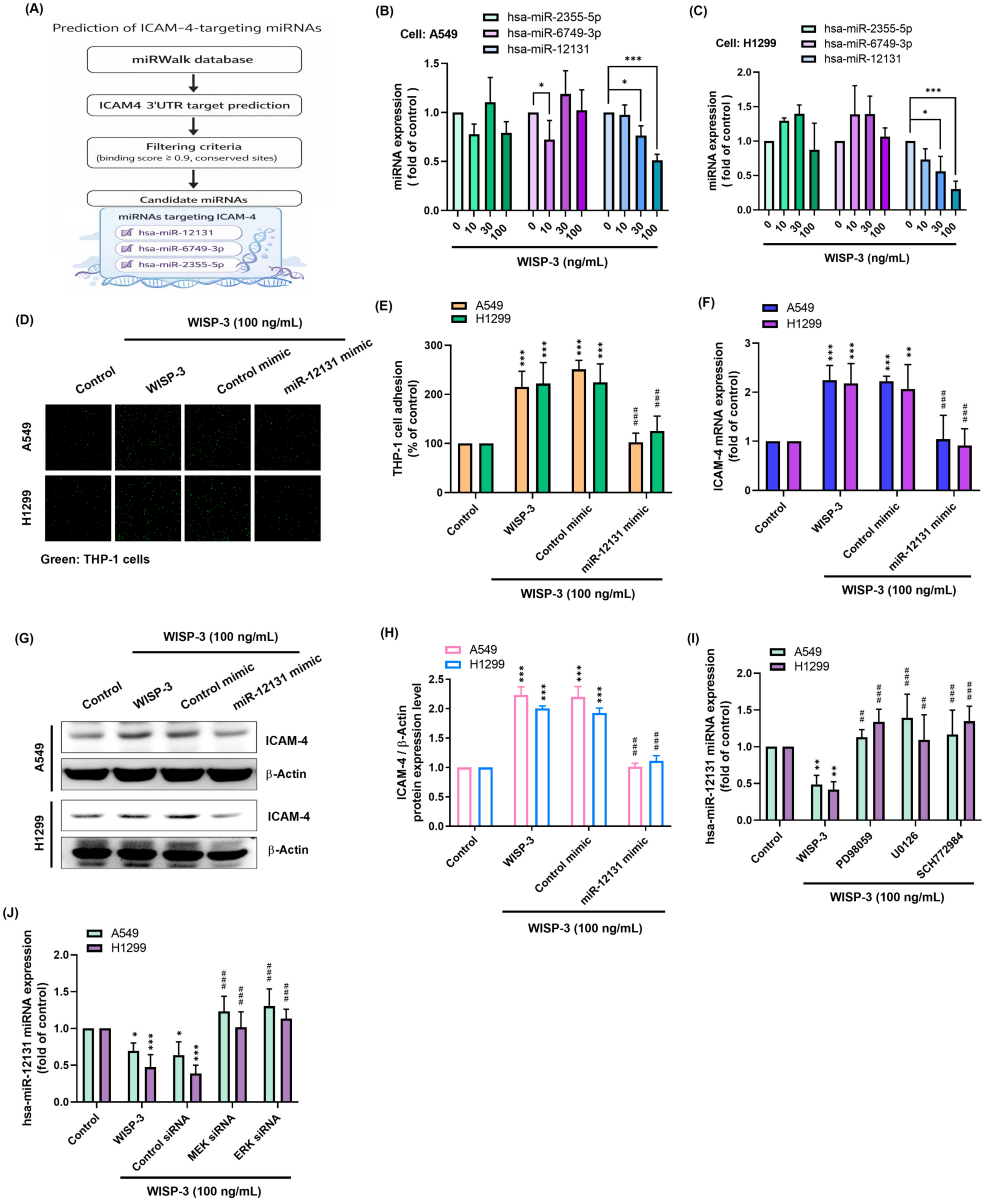
** WISP-3 promotes tumor-monocyte adhesion through suppression of miR-12131 in lung adenocarcinoma cells.** (A) Candidate miRNAs targeting ICAM-4 were predicted using the miRWalk database. (B&C) Expression of candidate miRNAs was measured by qPCR after treatment with WISP-3 (0, 10, 30, or 100 ng/mL) for 24 h. (D-H) Tumor-monocyte adhesion, ICAM-4 mRNA expression, and ICAM-4 protein expression were evaluated following transfection with control mimic or miR-12131 mimic and subsequent WISP-3 stimulation. (I&J) miR-12131 expression was measured following pharmacological inhibition or siRNA-mediated knockdown of MEK/ERK signaling. Data are presented as mean ± SD from at least three independent experiments. Statistical significance was defined as *P < 0.05, **P < 0.01, and ***P < 0.001 versus control; #P < 0.05, ##P < 0.01, and ###P < 0.001 versus WISP-3-treated group.

**Figure 5:**
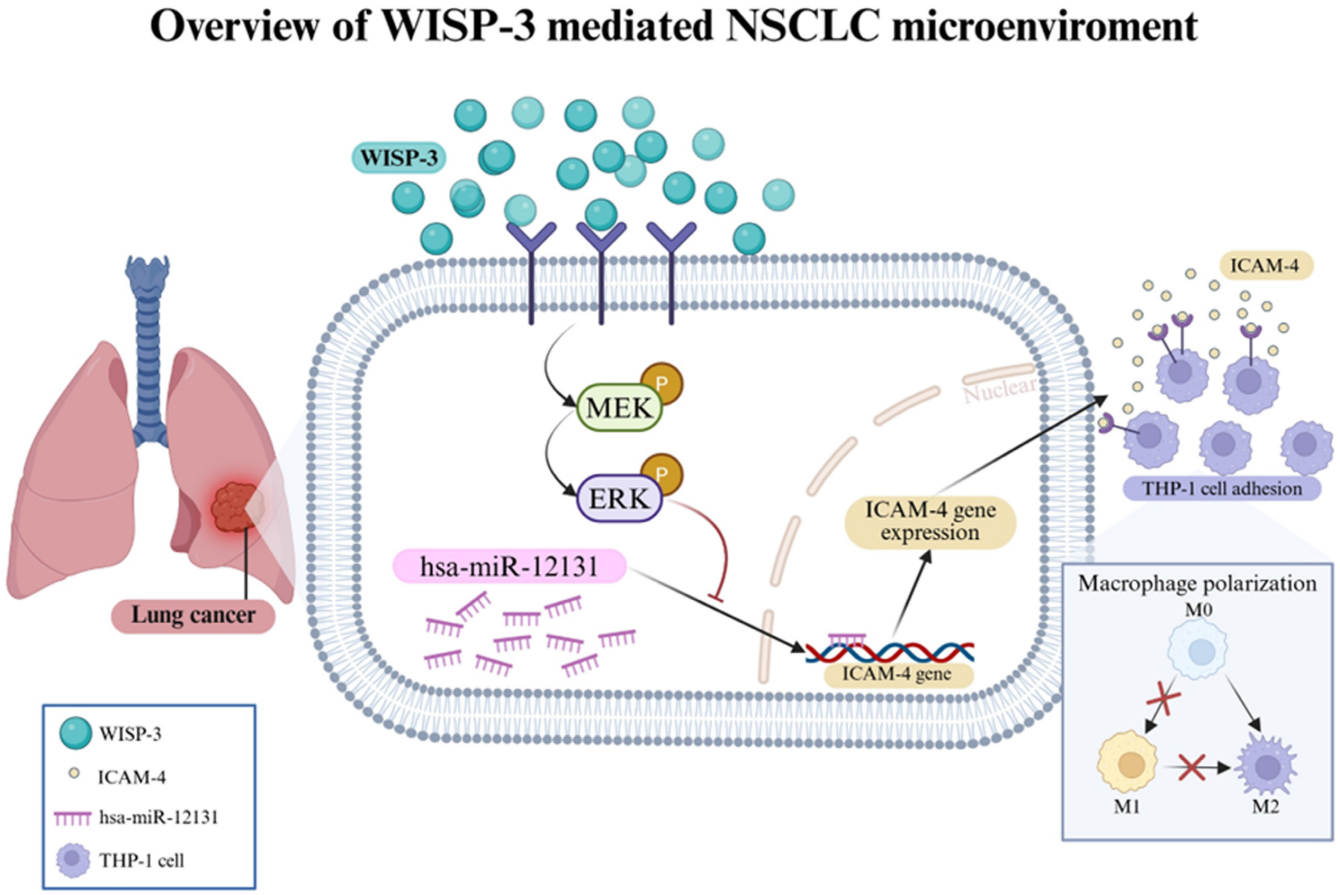
** Schematic illustration of the WISP-3-MEK/ERK-miR-12131-ICAM-4 signaling axis in lung adenocarcinoma.** WISP-3 activates MEK/ERK signaling, leading to suppression of miR-12131 expression. Downregulation of miR-12131 contributes to increased ICAM-4 expression, which is associated with enhanced tumor-monocyte adhesion in lung adenocarcinoma cells.

Because microRNAs are important post-transcriptional regulators of gene expression, we next asked whether miRNAs might participate in WISP-3-induced ICAM-4 expression. To this end, bioinformatics analysis was performed using the miRWalk database to predict miRNAs targeting the ICAM-4 3′UTR. Three candidate miRNAs, hsa-miR-2355-5p, hsa-miR-6749-3p, and hsa-miR-12131, were identified based on their predicted binding sites (Fig. 4A). We then examined whether these candidate miRNAs were affected by WISP-3 treatment. Among them, hsa-miR-12131 was consistently decreased in both A549 and H1299 cells in response to WISP-3, whereas the other candidates showed less evident changes (Fig. 4B&C). To further evaluate the functional role of miR-12131, cells were transfected with a miR-12131 mimic prior to WISP-3 stimulation. WISP-3 significantly increased THP-1 adhesion and ICAM-4 expression, whereas transfection with the miR-12131 mimic markedly attenuated WISP-3-induced THP-1 adhesion (Fig. 4D&E) and reduced WISP-3-induced ICAM-4 mRNA and protein expression in both A549 and H1299 cells (Fig. 4F-H). We next examined whether the reduction of miR-12131 by WISP-3 is regulated through MEK/ERK signaling. Treatment with PD98059, U0126, or SCH772984 significantly restored miR-12131 expression that had been decreased by WISP-3 (Fig. 4I). A similar effect was observed following knockdown of MEK or ERK (Fig. 4J). These results indicate that WISP-3 decreases miR-12131 through the MEK/ERK pathway, thereby promoting ICAM-4 expression and monocyte adhesion (Fig. 5).

## Discussion

Tumor-monocyte/macrophage interactions are increasingly recognized as critical determinants of tumor progression, metastasis, and immune evasion 5, 6. TAMs derived from circulating monocytes contribute to tumor growth and remodeling of the tumor microenvironment 20. Adhesion molecules play essential roles not only in physical cell-cell interactions but also in regulating immune cell recruitment and signaling within the tumor niche 21, 22. In this context, identifying signaling pathways that regulate tumor-monocyte adhesion may improve our understanding of how tumor cells interact with immune components of the microenvironment. Our findings suggest that WISP-3 participates in this process through coordinated regulation of MEK/ERK signaling, miR-12131 expression, and ICAM-4 expression.

WISP-3, a member of the CCN family of matricellular proteins, has been reported to exert context-dependent functions in cancer, acting as either a tumor suppressor or promoter depending on the cellular and microenvironmental context 23. CCN proteins are known to regulate diverse biological processes, including extracellular matrix remodeling, cell proliferation, and angiogenesis 24, 25. Although the role of WISP-3 in lung cancer remains incompletely defined, our findings extend its functional repertoire by demonstrating that WISP-3 regulates tumor-monocyte adhesion. Given that monocyte recruitment and differentiation into TAMs are critical steps in shaping the tumor microenvironment, the ability of WISP-3 to enhance tumor-monocyte adhesion suggests a potential role in immune modulation 26, 27.

The MEK/ERK signaling pathway is a central mediator of cellular responses to extracellular stimuli and is frequently dysregulated in non-small cell lung cancer 28, 29. Activation of this pathway has been implicated in multiple processes associated with tumor progression, including proliferation, survival, metastasis, and regulation of gene expression 30. Our findings support the notion that MEK/ERK signaling may also contribute to the regulation of adhesion-associated molecules involved in tumor-immune interactions. These observations are consistent with previous studies demonstrating that MAPK signaling can regulate both transcriptional responses and microRNA-mediated post-transcriptional programs in cancer cells 12, 31.

A key finding of this study is the identification of miR-12131 in WISP-3-mediated signaling. MicroRNAs are critical post-transcriptional regulators that control gene expression by targeting mRNAs and have been widely implicated in cancer progression and tumor-microenvironment interactions 32, 33. In lung cancer, miRNAs regulate multiple processes including cell proliferation, invasion, and immune modulation 32. However, the functional roles of many non-canonical miRNAs remain largely unexplored. Although hsa-miR-12131 has been annotated in public databases such as miRBase and is included in several target-prediction resources, its biological function remains poorly characterized, particularly in lung cancer and tumor-immune interactions. Our results demonstrate that miR-12131 may act as a regulatory intermediary linking MEK/ERK signaling to adhesion-associated gene expression. This observation expands the limited knowledge currently available regarding the biological function of miR-12131.

Another notable observation is the selective induction of ICAM-4. While ICAM-1 has been extensively studied in cancer and inflammation 34, the role of ICAM-4 in solid tumors remains poorly understood. Members of the ICAM family are known to mediate leukocyte adhesion and immune cell interactions 35, suggesting that ICAM-4 may contribute to tumor-immune crosstalk. The selective upregulation of ICAM-4 observed in this study indicates that WISP-3 activates a specific adhesion program rather than a generalized inflammatory response. Taken together, our findings support a model in which WISP-3 regulates tumor-monocyte adhesion through a MEK/ERK-miR-12131-ICAM-4 signaling axis in lung adenocarcinoma and provide additional insight into the molecular mechanisms underlying tumor-immune interactions.
